# Intra-operative and post-operative complications of endometriosis excision using the SOSURE approach — A single-surgeon retrospective series of 1116 procedures over 8 years

**DOI:** 10.52054/FVVO.16.3.030

**Published:** 2024-09-30

**Authors:** S Khazali, A Bachi, B Mondelli, K Fleischer, M Adamczyk, G Delanerolle, JQ Shi, X Yang, P Nisar, P Bearn

**Affiliations:** Centre for Endometriosis and Minimally Invasive Gynaecology (CEMIG London), HCA The Lister Hospital, Chelsea Bridge Road, London, United Kingdom, SW1W 8RH; Department of Obstetrics and Gynaecology, Ashford and St. Peter’s Hospital NHS Foundation Trust, Guildford Road, Chertsey, United Kingdom, KT16 0PZ; Digital Evidence Based Medicine Lab, Southern Health NHS Foundation Trust, Wessex Way, Colden Common, Winchester, United Kingdom, SO21 1WP; Department of Statistics and Data Science, Director Centre for Biostatistics, College of Science, Southern University of Science and Technology, 1088 Xueyuan Blvd, Nan Shan Qu, Shen Zhen Shi, Guang Dong Sheng, China, 518055; Department of Statistics and Data Science, College of Science, Southern University of Science and Technology, 1088 Xueyuan Blvd, Nan Shan Qu, Shen Zhen Shi, Guang Dong Sheng, China, 518055

**Keywords:** Complications, endometriosis, excision surgery, single surgeon, structured approach

## Abstract

**Background:**

Endometriosis surgery outcomes have been widely studied, yet heterogeneity in terminology and techniques persist.

**Objectives:**

This study focuses on the perioperative outcomes of a single surgeon using the same structured approach (SOSURE: Survey & Sigmoid mobilisation, Ovarian mobilisation, Suspension of uterus and ovaries, Ureterolysis, Rectovaginal and pararectal space development, Excision of all visible disease) and adheres to the recent standardised terminology proposed by international gynaecological and endometriosis societies.

**Materials and Methods:**

A quality improvement study was conducted retrospectively from January 2015 to January 2023. Data collection involved two databases: the National British Society for Gynaecological Endoscopy (BSGE) database and a more comprehensive locally kept database. The methodology also integrated four endometriosis staging systems.

**Main outcome measures:**

Intra-operative and post-operative complication rates.

**Results:**

Between 2015 and 2023, 1047 women underwent 1116 endometriosis procedures in various UK hospitals with S.K. as primary surgeon. Exclusions totalled 20 due to missing records and specific surgical criteria. The rate of major post-operative complications (Clavien-Dindo grade 3a and 3b) was 1.5% and minor post-operative complications (Clavien-Dindo grade 1 and 2) were seen in 13.8%. No Clavien-Dindo grade 4 or 5 complications were noted.

**Conclusion:**

Our study has shown a low complication rate in endometriosis surgery, despite increasing complexity of surgical cases. This is likely attributed to the surgeon’s learning curve, high surgical volume and adherence to a structured approach.

**What's new?:**

Our study demonstrates the learning curve of a surgeon over the course of 8 years. This series involved more than 1000 patients and to our knowledge, is the first to report the complexity of the casemix using four different endometriosis staging systems.

## Introduction

Numerous studies have examined outcomes after endometriosis surgery. However, there are significant variations in both data and terminology. For instance, some researchers describe “shaving” as the removal of the endometriotic nodule without penetrating deeper than the rectal muscularis, and without the use of sutures. Others define it to encompass procedures that excise part of the muscularis layer, necessitating manual suturing (Bendifallah et al., 2021). The term “rectovaginal fistula” has also seen varied definition (Bendifallah et al., 2021; Revised American Society for Reproductive Medicine Classification of Endometriosis: 1996, 1997; International Working Group of AAGL, ESGE, ESHRE and WES et al., 2021). Notably, most research has aggregated data from various surgeons employing different techniques. Only a handful of studies have focused on outcomes from a single surgeon maintaining a consistent philosophy and surgical approach.

This study was initiated to evaluate complication rates of procedures performed by a single surgeon over an eight-year span, employing the same structured surgical approach consistently. For this study, we have adopted the nomenclature recommended by the consensus of the International Working Group of AAGL, European Society for Gynaecological Endoscopy (ESGE), European Society of Human Reproduction and Embryology (ESHRE), and the World Endometriosis Society (WES) (International Working Group of AAGL, ESGE, ESHRE and WES et al., 2021) and used rASRM (Revised American Society for Reproductive Medicine Classification of Endometriosis: 1996, 1997), AAGL (Abrao et al., 2021), #Enzian (Keckstein et al., 2021) and VNESS systems for classification of endometriosis.

## Materials and methods

### Study design and patient selection

This quality improvement study involved a retrospective analysis of data drawn from a single surgeon’s (S.K.) practice spanning from 1st January 2015 to 1st January 2023. No ethical approval was required. We included all patients diagnosed with endometriosis who underwent laparoscopic, robotic-assisted, or open surgery performed by S.K. in the UK during this period. Exclusions were made for patients:

1. Who underwent surgical excision of abdominal wall or extra-pelvic endometriosis without concurrent pelvic disease.
2. Lacking essential medical documentation, such as surgical notes.

From 2013 to 2016, S.K. periodically visited Iran to teach and perform endometriosis surgery. During these visits, S.K. performed 462 endometriosis procedures, including 92 segmental bowel resections. To avoid publication duplication, these cases were not incorporated into this study, as the majority have been recently included in another publication (Khazali et al., 2019).

### Clinical Assessment

All patients underwent a clinical examination conducted by a gynaecologist specialised in endometriosis, and a transvaginal ultrasound scan (TVUSS). Those suspected of having rectovaginal endometriosis also had a pelvic magnetic resonance imaging (MRI) and were reviewed in a multidisciplinary meeting consisting of gynaecologists, radiologists, colorectal surgeons, urologists, and a specialist nurse.

### Surgical Technique

Patients primarily underwent either laparoscopy or robotic-assisted laparoscopy. Those diagnosed with rectovaginal endometriosis received bowel preparation a day prior to their surgery, complemented by antibiotic prophylaxis before anaesthesia induction.

For laparoscopy, a 10mm umbilical port and two or three secondary 5mm ports were inserted, one in the midline and one on left (and right) iliac fossae. The primary energy devices utilised were the Harmonic® scalpel and Thunderbeat®.

For the robotic-assisted surgeries, introduced to the author’s practice in March 2022, the Da Vinci Surgical System Xi was utilised. An umbilical port was designated for the laparoscope. Two or three 8mm ports were aligned on the same plane for the robotic arms, supplemented by a 5mm or 12mm assistant port.

The SOSURE approach (Fleischer et al., 2021), described previously by the authors was incorporated. SOSURE is a mnemonic describing steps for the normalisation of anatomy and optimising surgical access prior to the excision of the disease. This approach was utilised consistently for all cases throughout the study period.

The SOSURE steps are ‘Survey and Sigmoid mobilisation’, ‘Ovarian mobilisation’, ‘Suspension of ovaries and uterus’, ‘Ureterolysis’, ‘Rectovaginal and pararectal spaces development’ and ‘Excision of all visible disease’. All steps may not be necessary and the order by which these are performed can vary from case to case.

The sigmoid colon is first mobilised from the sidewall, providing access to the left adnexa and aiding left ureterolysis. The ovaries are mobilised if adhered. Using a rectus sheath closure device (Endo Close®), the uterus and ovaries are suspended to the anterior abdominal wall. This not only frees the surgical assistant from acting primarily as a ‘retractor’ but also aids in tissue dissection and maintaining a clear surgical view. In robotic-assisted procedures, this step might obviate the need for the fourth robotic arm. Additionally, the suspension of the uterus, in our experience, offers a better and more stable traction and anteversion of the uterus compared to a uterine manipulator.

Endometriomas were managed by excision, drainage, ablation, or alcohol sclerotherapy (introduced in 2021) depending on the circumstances. The treatment of endometriomas were mainly performed at the beginning of the surgery prior to suspension, and on occasion the ovarian suspension was left in situ until day five post-operative to reduce the risk of adhesion formation. Partial vaginectomy was done in cases where endometriosis had infiltrated the vaginal fornix, and an appendicectomy was performed if signs of involvement were present.

We have used the terms and definitions for treatments and interventions in line with the multi- society consensus on endometriosis terminology (International Working Group of AAGL, ESGE, ESHRE and WES et al., 2021). However, in this consensus, the definition of shave was not reached and as such, we have defined shaving as the excision of bowel serosal and subserosal endometriosis. The procedure is termed a “partial-thickness discoid excision” when the disease has infiltrated the superficial muscularis layer of the bowel and following excision, the defect required reinforcement sutures (i.e. closure of a muscularis defect without a mucosal defect in the bowel wall). In contrast, “full-thickness discoid excision” was characterised by either opening the anterior wall of the rectum, excising the disease and repairing the defect using two layers of resorbable stitches or employing a trans- anal circular stapler. This technique was reserved for nodules measuring less than 3cm. For larger nodules (usually exceeding 3cm) or instances of multifocal disease, we opted for a segmental bowel resection. For all cases with concomitant opening of the bowel and vagina, omentoplasty was performed to reduce the risk of rectovaginal fistula.

After any bowel procedure, its integrity was assessed using either a Michelin test or rigid sigmoidoscopy. All specimens were sent for histopathologic analysis.

### Data collection

Data was gathered from two distinct sources: the official National British Society for Gynaecological Endoscopy (BSGE) database and S.K.’s “local database”. Both databases are filled on or soon after the date of surgery. The BSGE database focuses on patients with rectovaginal endometriosis (where pararectal spaces had to be entered), capturing patient symptoms, quality of life, surgical details, and major complications. In contrast, the local database covered all procedures, offering an expansive overview of surgical findings, procedures, and a wider range of complications. To ensure data accuracy, especially concerning post-operative complications, a dedicated endometriosis nurse oversaw updates and acted as the primary patient liaison in our routine practice. Standard follow-up was conducted within 6 weeks post-surgery through phone or in-person consultations, for both patients travelling from within and outside United Kingdom, with patients discharged as suitable. However, patients receiving bowel surgery received extended monitoring at intervals of 6, 12, and 24 months via online questionnaires.

In 2022, we revised the local database to incorporate the new terminology for endometriosis (International Working Group of AAGL, ESGE, ESHRE and WES et al., 2021) and to include the following four endometriosis classification systems: the 2021 American Association of Gynecologic Laparoscopists (AAGL) classification system (Abrao et al., 2021), revised American Society of Reproductive Medicine (rASRM) classification system (Revised American Society for Reproductive Medicine Classification of Endometriosis: 1996, 1997), #ENZIAN classification system (Keckstein et al., 2021) and the Visual Numeric Endometriosis Scoring System (VNESS).

VNESS is a staging system conceptualised by the author which divides the pelvis into nine anatomical compartments (Supplementary Figure 1): left adnexa (LADN), left pelvic sidewall (LPSW), left uterosacral ligament (LUSL), uterovesical fold (UVF), vaginal and rectovaginal space (VAG), pouch of Douglas and rectum (RECT), right uterosacral ligament (RUSL), right pelvic sidewall (RPSW), right adnexa (RADN). Each compartment is given a disease severity score, ranging from zero to four (0 = no macroscopic evidence endometriosis, 1 = superficial endometriosis, 2 = deep endometriosis with no adhesions or with filmy adhesions to surrounding structures, 3 = deep endometriosis with dense adhesions to surrounding structures, 4 = deep endometriosis invading into surrounding structures). As such, VNESS consists of 9 numbers which are written from left to right, in an order resembling the pelvic survey during a diagnostic laparoscopy, with the first number representing the left adnexa and the last number representing the right adnexa. This aids in visualising the severity of disease in each pelvic compartment. A validation study for VNESS has been submitted for publication and is undergoing peer-review.

In 2022, following the update of the local database, electronic hospital records of all patients were retrospectively assessed by clinical fellows to supplement missing information and bolster data accuracy. Two gynaecologists (S.K. and A.B.) further reviewed the complication data to correct any discrepancies. The examined records encompassed operation notes, surgical images, inpatient documentation, and follow-up consultation letters. Patients with missing crucial data (such as those missing both operation notes and images) that impeded precise data capture were excluded. To mitigate bias, we cross-referenced our local database for any complications noted in these patients.

The intra-operative adverse events were classified according to the ClassIntra classification system (Dell-Kuster et al., 2020) (Supplementary Table I). The post-operative complications were classified according to the Clavien-Dindo classification system (Dindo et al., 2004). We classified grade 1 and 2 complications as minor and grade 3 and 4 complications as major.

### Reliability Analysis

We used Cronbach’s alpha coefficient to assess the reliability of data collected from four endometriosis classification systems: AAGL, rASRM, #ENZIAN, and VNESS. The Cronbach’s alpha ranges from 0 to 1, and a higher value indicates greater internal consistency among the associated items. In most social science research situations, a reliability coefficient of 0.7 or higher is considered “acceptable.” We employed SPSS software to calculate the Cronbach’s alpha for these four systems.

## Results

Between January 2015 to January 2023, 1047 women underwent 1116 procedures for endometriosis across both the public and private sectors in the UK (n=387 and 729 respectively).

20 patients were excluded from the analysis; 18 (1.61%) had incomplete or missing crucial hospital records and 2 (0.18%), had surgical excision of abdominal wall endometriosis only, without any pelvic endometriosis. Supplementary Table II shows the number and proportion of patients excluded due to missing or incomplete hospital records. None of the excluded patients recorded any intra-operative or post-operative complications.

### Surgical volume and complexity

**Figure 1:**
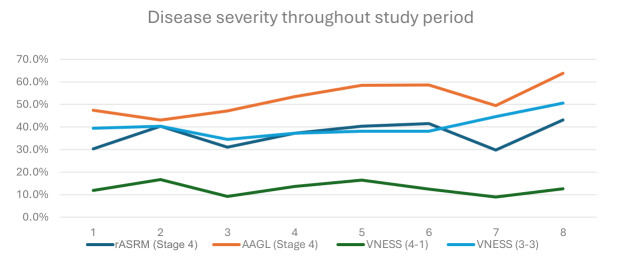
Severity of endometriosis cases throughout the study period. * VNESS (4-1): VNESS score 4 in 1 or more compartment, VNESS (3-3): VNESS score 3 in 3 or more compartment.

The mean age was 37 (range 17 – 60). Out of the 1116 surgical procedures, 571 bowel procedures (51.2%) were performed. The number of surgical procedures and bowel procedures each year is presented in Table I. The severity of endometriosis cases is presented in Figure 1. The complexity of the caseload showed an overall increase throughout the study period, with a peak in year 2019. In 2020 and 2021, there was a drop in the surgical volume due to the Covid-19 pandemic when all elective surgeries were either temporarily suspended or scaled down.

**Table I:** Surgical procedures and bowel procedures performed each year.

	2015	2016	2017	2018	2019	2020	2021	2022	Total
No. of Endometriosis cases	76	72	87	161	226	152	168	174	1116
Discoid Excision (full-thickness)1--111149	1	-	-	1	1	1	1	4	9
Discoid Excision (partial-thickness)	3	4	2	5	8	10	12	22	66
Segmental Bowel resection	3	10	6	11	8	8	3	11	60
Shave of Rectovaginal Endometriosis	24	14	33	69	101	66	65	64	436
Ileostomy	3	3	-	1	-	-	-	-	7

### Procedure route and caseload complexity

Table II shows the intra-operative data. 99.4% of procedures were completed laparoscopically (n=1051) or by robotic-assisted laparoscopy (n=59). The latter was introduced into our practice in March 2022. One case was performed through an open route for a myomectomy for an 18-week sized uterus and shave of rectovaginal endometriosis.

**Table II:** Intraoperative data.

Intraoperative findings				No.	%
Route of procedure				No.	%
	Laparoscopy			1051	94.2%
	Robotic assisted			59	5.3%
	Laparoscopy converted to open			5	0.4%
	Open			1	0.1%
Staging				No.	%
	ASRM staging				
		Stage 1		344	30.8%
		Stage 2		228	20.4%
		Stage 3		101	9.1%
		Stage 4		434	38.9%
	AAGL staging			No.	%
		Stage 1		151	13.5%
		Stage 2		55	4.9%
		Stage 3		30	2.7%
		Stage 4		603	54.0%
	*VNESS staging			No.	%
		VNESS (4-1)		144	12.9%
		VNESS (3-1)		581	52.1%
		VNESS (3-3)		456	40.9%
		VNESS (2-1)		549	49.2%
		VNESS (1-1)		458	41.0%
	ENZIAN staging			No.	%
		Peritoneum			
			P1	290	26.0%
			P2	178	15.9%
			P3	65	5.8%
		Ovary**			
			O1	113	10.1%
			O2	168	15.1%
			O3	49	4.4%
		Tube**			
			T1	186	16.7%
			T2	75	6.7%
			T3	332	29.7%
		A (Rectovaginal space, vagina, retrocervical area)			
			A1	4	0.4%
			A2	257	23.0%
			A3	416	37.3%
		B (Sacrouterine ligament, cardinal ligament, pelvic sidewall)**			
			B1	28	2.5%
			B2	316	28.3%
			B3	598	53.6%
		C (Rectum)			
			C1	18	1.6%
			C2	309	27.7%
			C3	347	31.1%
		FA (adenomyosis)		452	40.5%
		FB (bladder)		174	15.6%
		FI (intestine)		9	0.8%
		FU (ureter)		55	4.9%
Bowel procedures				No.	%
	Shave			436	39.1%
	Partial-thickness discoid excision			66	5.9%
	Full-thickness discoid excision			9	0.8%
	Segmental bowel resection			60	5.4%
	Primary ileostomy			7	0.6%
	Emergency ileostomy			4	0.4%
	Appendicectomy			50	4.5%
Other surgical procedures				No.	%
	Hysterectomy			216	19.4%
	Partial vaginectomy			63	5.6%

* VNESS (4-1): VNESS score 4 in 1 or more compartment, VNESS (3-1): VNESS score 3 in 1 or more compartment, VNESS (3-3): VNESS score 3 in 3 or more compartments, VNESS (2-1): VNESS score 2 in 1 or more compartment, VNESS (1-1): VNESS score 1 in 1 or more compartment

** For paired organs, the higher severity has been chosen for this table.

Our local database was designed to calculate four different endometriosis staging/classification systems using the raw surgical data. This enabled us to have rASRM, AAGL, #Enzian and VNESS for all cases (Table II).

### Reliability analysis

As shown in Table III, all of the Cronbach’s alpha coefficients of these four systems exceeded 0.8. Notably, the VNESS system had a Cronbach’s alpha of 0.94, substantially higher than the other systems, suggesting that its items have relatively high internal consistency.

**Table III:** Reliability Analysis for the Four Endometriosis Classification Systems.

Endometriosis Classification System	The Number of Items	Cronbach’s Alpha
AAGL	15	0.879
rASRM	8	0.828
ENZIAN	9	0.876
VNESS	9	0.94

### Primary ileostomy

A primary ileostomy was created in seven cases, all of which were performed in the first four years of the study period as described in Table IV. The rate of primary ileostomy following segmental bowel resection, and all bowel procedures was 10% (6/60) and 1.2% (7/571) respectively. Four of these patients had concomitant partial vaginectomy and segmental bowel resection. All ileostomies were reversed three to six months after the primary procedure, without complications. None of the patients with primary ileostomies developed anastomotic insufficiencies or rectovaginal fistula.

**Table IV:** Rate of ileostomy.

Ileostomy	All procedures	All bowel procedures	Segmental bowel resections
Total primary ileostomy (2015-2022)	0.6% (7/1116)	1.2% (7/571)	10% (6/60)
Primary Ileostomy (2015-2018)	1.8% (7/396)	3.8% (7/186)	20% (6/30)
Primary Ileostomy (2019-2022)	-	-	-
Total emergency Ileostomy (2015 – 2022)	0.6% (4/720)	0.7% (4/571)	3.3% (1/30)
Emergency Ileostomy (2015-2018)	-	-	-
Emergency Ileostomy (2019-2022)	0.6% (4/720)	1% (4/385)	3.3% (1/30)

### Intra-operative adverse events

**Figure 2:**
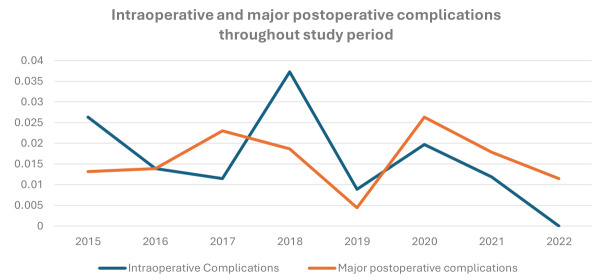
The rate of intraoperative and major postoperative complications during the study period.

Table V presents the intra-operative adverse events. The rate of intra-operative and major post-operative complications remained stable throughout the study period, as shown in Figure 2. The rate of intra-operative adverse events was 1.5% (17/1116). All adverse events were ClassIntra grade 2. The rate of intra-operative adverse events showed a general decline from 2.6% in 2015 to 0% in 2022. There were three bowel serosal injuries and five unintended enterotomies, three bladder injuries and four ureteric injuries. All enterotomies and bladder injuries were managed by suturing over the defect in two layers and all ureteric injuries were managed with ureteric stenting. All patients had an uneventful post-operative recovery. There were no recorded vascular injury or intra-operative haemorrhage requiring blood transfusion.

**Table V:** Intraoperative adverse events.

Intraoperative adverse events		No.	%
Bowel injury		8	0.7%
	Bowel serosal injury requiring suture	5	0.4%
	Enterotomy	3	0.3%
Bladder injury		3	0.3%
Ureteric injury		4	0.4%
Uterine perforation		2	0.2%
Total		17	1.5%

### Post-operative complications

The rate of minor and major post-operative complications was 13.8% (154/1116) and 1.5% (17/1116) respectively. Of the 17 patients who had major post-operative complications, 2 had Clavien- Dindo grade 3a and 15 grade 3b. The overall rate of post-operative complications was 15.3% (171/1116).

The rate of major post-operative complications did not show any change throughout the study period. There were no recorded major complications in patients who underwent robotic surgery and there were no grade 4 or grade 5 complications.

### Minor post-operative complications

The most frequent minor complications were urinary tract infection (3%), followed by wound infection (2.5%) and post-operative voiding dysfunction (2.5%). All women with post-operative voiding dysfunction were managed with reinsertion of a urinary catheter. Only two patients (0.2%) further required intermittent self-catheterisation, one for 3 months and another one for 4 months. Six cases of post-operative blood transfusion (0.5%) were noted in our study.

### Major post-operative complications

Table VI presents the major post-operative complications. The rate of major post-operative complications in those who underwent segmental bowel resection, and all bowel surgery was 6.7% (4/60) and 2.1% (12/571) respectively, as described in Table VII. The detailed description of each grade 3b complication is shown in Supplementary Table III.

**Table VI:** Major postoperative complications.

Major postoperative complications		No.	%
Grade 3a		2	0.2%
	Bowel leak managed with IR drainage	1	0.1%
	Pelvic abscess requiring IR drainage	1	0.1%
Grade 3b		14	1.3%
	Anastomotic stricture	1	0.1%
	Bowel leak	2	0.2%
	Pelvic abscess requiring surgical drainage	1	0.1%
	Pelvic haematoma requiring surgical drainage	2	0.2%
	Rectovaginal fistula	2	0.2%
	Exploratory laparoscopy	1	0.1%
	Urinary tract fistula	1	0.1%
	Bleeding from port site	1	0.1%
	Haematuria requiring surgical intervention	1	0.1%
	Intraabdominal bleeding requiring surgical intervention	2	0.2%

**Table VII:** Major post-operative complications specific to bowel procedures.

Major post-operative Complications (3a above)		All procedures	All bowel procedures	Segmental bowel resection
Total			1.5% (17/1116)	2.1% (12/571)	6.7% (4/60)
	Grade 3a		0.2% (2/1116)	0.4% (2/571)	3.3% (2/60)
		Bowel leak managed with IR drainage	0.1% (1/1116)	0.2% (1/571)	1.7% (1/60)
		Pelvic abscess requiring IR drainage	0.1% (1/1116)	0.2% (1/571)	1.7% (1/60)
	Grade 3b		1.3% (15/1116)	1.8% (10/571)	3.3% (2/60)
		Anastomotic stricture	0.1% (1/1116)	0.2% (1/571)	1.7% (1/60)
		Bowel leak	0.2% (2/1116)	0.4% (2/571)	-
		Pelvic abscess requiring surgical drainage	0.1% (1/1116)	0.2% (1/571)	-
		Pelvic haematoma requiring surgical drainage	0.2% (2/1116)	-	-
		Rectovaginal fistula	0.2% (2/1116)	0.4% (2/571)	1.7% (1/60)
		Exploratory laparoscopy	0.1% (1/1116)	-	-
		Urinary tract fistula	0.1% (1/1116)	-	-
		Bleeding from port site	0.1% (1/1116)	0.2% (1/571)	-
		Haematuria requiring surgical intervention	0.1% (1/1116)	0.2% (1/571)	-
		Intraabdominal bleeding requiring surgical intervention	0.2% (2/1116)	0.2% (1/571)	-

There were four bowel leaks, two following segmental bowel resections, one following shave and one following partial-thickness discoid excision. One case was managed with antibiotics in view of the patient being clinically well and the small size of collection measuring only 3mm. One was managed with CT-guided percutaneous drainage and two with laparoscopy and insertion of a diverting stoma. In one of these cases, the patient initially presented with a pelvic abscess which was managed with surgical drainage. This patient then represented four days later with peritonitis and a CT scan confirmed a bowel leak. The patient required a third surgery for the insertion of a diverting stoma.

There were two cases of rectovaginal fistula (0.2%), one following rectal shaving and hysterectomy and one following segmental bowel resection and partial vaginectomy. In our study, the rate of rectovaginal fistula following bowel surgery, segmental bowel resection and in patients who had concomitant rectal and vaginal sutures (patients who underwent full-thickness discoid excision or segmental bowel resection, along with partial vaginectomy or hysterectomy) were 0.4% (2/571), 1.7% (1/60) and 2.9% (1/35) respectively.

In total, 11 ileostomies were created during the study period. Seven were primary ileostomies, all of which occurred in the first half of the study period. The remaining four were diverting stomas carried out during emergency surgery for patients with bowel leakage or rectovaginal fistula, all of which occurred in the second half of the study period.

## Discussion

This study represents data on a single surgeon which reflects consistent philosophy of care and limits heterogeneity in surgical practice and decision-making protocol. It is important to note that this consistency did not mean a rigid standstill as throughout the study period, S.K.’s approach and skills as well of that of the multidisciplinary team evolved gradually as the team adopted new techniques such as alcohol sclerotherapy, robotic assisted surgery, transanal discoid excision for larger nodules and so on. Another example of this gradual evolution in practice is the fact that in the second half of the study period, no planned ileostomies were done.

Our study showed a low rate of intra- operative complications and major post-operative complications. A description of cases converted to open is seen in Supplementary Table IV. We did not include conversions to laparotomy under intra- operative complications. Three of the cases occurred in women with ureteric endometriosis and they were planned conversions by the urological team who only performed ureteric reimplantation or end- to-end anastomosis through an open approach. In Iran, the author performed six laparoscopic ureteric reimplantation, jointly with a transplant surgeon who was observing. However, this set up could not be replicated in the UK with the urology team available at the time. The other case for conversion occurred due to a malfunctioned circular stapler and as such, a subumbilical incision was made to complete the segmental bowel resection.

Kondo et al. (2011) investigated 568 women who underwent surgery for deep endometriosis (DE) and reported an overall rate of major intra- operative and post-operative complications of 1.05% and 4.6% respectively. The rate of major post-operative complications further increased to 9.3% in patients undergoing any type of rectal surgery (Kondo et al., 2011). Our rates of major complications following all types of bowel surgery and segmental bowel resection were 2.2% and 6.6% respectively. Previous studies have compared the complication rates between the three different surgical procedures (Abo et al., 2018; Bafort et al., 2020; Bendifallah et al., 2021; Byrne et al., 2018; Donnez and Roman, 2017). Abo et al. (2018) evaluated 364 patients undergoing bowel surgery and found the mean rate of Clavien-Dindo 3b complications to be 11.8%, at which more than half were in the segmental bowel resection arm, with a rate of 20.9%. A recent systematic review by Bendifallah et al. (2021) reported an overall complication rate which ranged from 2.2% to 9.9%, with the mean complication rate associated with shaving, disc excision, and segmental resection to be 2.2%, 9.7%, and 9.9%, respectively. Whilst these findings support a more conservative surgical approach in the management of bowel endometriosis, it is important to note that disease in these three groups may not be similar in terms of extent and severity. Other factors that can contribute to these complication rates include the overall difficulty of the surgery itself, the need for concomitant vaginal or urologic surgery, the presence of low nodules leading to risk of denervation and low anastomotic line.

Rectovaginal fistula is one of the most debilitating post-operative complications. There were two recorded fistulas in our study, one following shave and one following segmental bowel resection. The rate of fistula following segmental bowel resection was 1.6%, which is comparable to the existing literature (Balla et al., 2018; Ceccaroni et al., 2023; Malzoni et al., 2016; Renner et al., 2017; Ruffo et al., 2010; Ruffo et al., 2012). A systematic review by Balla et al. (2018) has observed a rate of fistula to be 2.4% following segmental bowel resection whilst a study by Malzoni et al. (2016) which evaluated 248 segmental bowel resections for endometriosis reported the rate of fistula to be 2.4% and all cases of fistula occurred in patients with very low anastomosis, concomitant vaginal suture and no protective ileostomy. In a recent study by Ceccaronni et al. (2023), the rate of fistula in 3050 segmental resection was reported to be 1.9%. These figures are comparable to that of our study. On the other hand, Mangler et al (2014) reported no incidence of fistula in 71 cases of bowel resection.

One risk factor for rectovaginal fistula is the apposition of two suture lines (Belghiti et al., 2014; Kondo et al., 2011; Roman et al., 2020), where there is concomitant vaginectomy or hysterectomy and discoid resection or segmental bowel resection. However, most authors do not specifically report the rate of fistula in this subgroup. In our analysis, the rate of fistula in this cohort is further increased to 2.9%. Belghiti et al. (2014) reported rate of fistula to be 13% in patients requiring colorectal resection and partial colpectomy. Conversely, Ceccaroni et al. (2021) reported no incidence of rectovaginal fistula following 371 discoid resection and, in this study, 39.6% of the cohort had concomitant vaginal resection or hysterectomy. The author hypothesised that this aspect could be related to precise preoperative assessment of the rectal nodule as well as proper intra-operative surgical decision making in terms of vaginal suturing and protective ileostomy, if necessary (Ceccaroni et al., 2021).

In our study, for all cases of concomitant bowel and vaginal surgery, omental flap was systematically placed between rectal and vaginal repair sutures. Some surgeons believe omentoplasty can reduce the risk of fistula by increasing neovascularisation (Boudy et al., 2020). This technique involves transposition of a vascularised pedicle of omentum to cover the anastomosis, however, this is not always possible due to its anatomical characteristics and difficulties in releasing sufficient omentum (Boudy et al., 2020). Other methods of interposing extra tissue between the rectal and vaginal staple lines include the use of a biological mesh, placing a row of imbricating stitches in the seromuscularis layer over the staple line or applying an organic plate or prosthesis on the vaginal defect (Boudy et al., 2020; Redwine, 2004).

A recent study by Hudelist et al. (2022) has demonstrated a lower complication rate in high volume centres. Table VIII compares our complication rates in patients undergoing bowel procedures with the three categories of centres reported by Hudelist et al. (2022). Throughout the study period, we have observed a growth in the volume of surgical activity. The rate of intra- operative and major post-operative complications remained stable throughout. This is likely due to the progressive increase in surgical skill being offset by the rise in complications resulting from performing more complex procedures. This is demonstrated in the increasing trend in the proportion of cases of severe endometriosis, classified as stage 4 endometriosis based on both the AAGL and rASRM classification. This finding aligns with a study conducted by Roman et al. (2021) which demonstrated no reduction in the rate of fistula among patients undergoing surgery for bowel endometriosis over a 15-year period. The author emphasised the need for caution when interpreting crude complication rates, and the percentage of patients undergoing challenging procedures which is known to be associated with higher expected complication rates should be taken into consideration.

**Table VIII:** Major complication rates from Hudelist et al. (2022) compared to our data.

	Study from Hudelist et al. (2022)			Our study
	Volume of activity/2 years			
	<40 (8 centers)	40-59 (6 centers)	≥ 60 (5 centers)	
Complication rates	n=190	n=274	n=473	
Leakage	4.21% (8/190)	2.19% (6/274)	1.06% (5/473)	0.53% (3/571)
Fistula	2.11% (4/190)	1.46% (4/274)	0.42% (2/473)	0.35% (2/571)
Haemorrhage	1.58% (3/190)	2.19% (6/274)	1.27% (6/473)	0.35% (2/571)
Pelvic abscess	0% (0/190)	1.09% (3/274)	0.21% (1/473)	0.18% (1/571)
Total	7.89% (15/190)	6.93% (19/274)	2.96% (14/473)	1.40% (8/571)

In our study, the rate of primary ileostomy was 11.1%, 10% and 1.3% following full-thickness discoid excision, segmental bowel resection and all bowel procedures respectively. The rate of primary ileostomy varies in the literature. Braund et al. (2021) reported the use of a primary stoma in 46.7% of patients undergoing discoid resection and 44.4% of patients undergoing segmental bowel resection. Similarly, Abo et al. (2018) showed that 55% and 48.2% of patients undergoing discoid resection and segmental resection respectively required a stoma whilst a recent study by Ceccaroni et al. (2021) reported an ileostomy rate of 27.4% in segmental bowel resection. Conversely, Malzoni et al. (2016) showed a very low rate of protective ileostomy of 1.6% in 248 women undergoing segmental bowel resection. In our study, all primary ileostomies were placed in the first four years of the study period. There were no primary ileostomies performed in the second half of the study period despite increasing complexity of cases performed. This change represents the increasing confidence and learning curve of the primary surgeon and colorectal surgeon. However, it is also important to note that all complications requiring emergency ileostomy (anastomotic leak and rectovaginal fistula) occurred in the last four years of the study period, when no primary ileostomy was placed. There is lack of strong evidence supporting the role of protective defunctioning stoma in reducing the occurrence of post-operative digestive tract complications following colorectal resection for endometriosis. Belghiti et al. (2014) showed no significant difference in the rate of fistula observed between patients with partial colpectomy and low colorectal anastomosis with and without primary stoma (p=0.39). Similarly, Roman et al. (2020) compared two groups of women with concomitant vaginal and rectal sutures with different rates of preventative stoma, 32.2% and 8.6%. The author showed the rates of fistula recoded in the two groups were 9.2% and 11.1% (p=0.80) (Roman et al., 2020). It is important to consider the morbidity associated with a protective ileostomy. In a study involving 163 women with colorectal endometriosis with a temporary stoma, 1 in 5 women presented with minor complications related to abdominal wall stoma scar such as infection, dehiscence, delayed healing and stoma prolapse and 8.6% of women had more serious complications such as leakage, hernia, bowel obstruction syndrome and haemoperitoneum after stoma closure. This study also showed that such complications led to a second surgery in 8% of cases (Bonin et al., 2019). In our study, there were no stoma-related complications, however this data cannot be extrapolated due to very small numbers.

To our knowledge this is the first study which uses a tool to calculate and report four endometriosis staging systems. This is also the first study to incorporate VNESS. A validation study for VNESS using 93 video clips reviewed by 50 gynaecologists has been concluded and is currently under peer review. Since the validation study remains unpublished, the VNESS scores in our study should be interpreted only as a general indicator of complexity across the nine pelvic compartments.

Our team included a dedicated endometriosis specialist nurse who meticulously tracked patient progress and served as a direct point of contact for clinical inquiries. Our comprehensive database carefully documented both intra-operative and post-operative details. When updating the database, clinical fellows cross-referenced the data against hospital records to rectify any discrepancies. Ultimately, all complications underwent a review by the author in collaboration with another gynaecologist. While these measures demanded additional time, they significantly enhanced the accuracy of our data and the credibility of our findings.

One limitation of this study concerns the lack of data on patient demographics such as body mass index (BMI), details of previous surgery, bowel function at baseline and post-operative and certain intra-operative data such as operating time, height of colorectal nodule level or the suture or anastomosis in relation to the anal verge, which may influence the incidence of major post-operative complications. As data was collected retrospectively, some information was not routinely collected. The other limitation of our study is that a number of patients had to be excluded from the analysis due to incomplete medical records. However, to minimise the risk of bias, we crosschecked these patients against our local database to ensure no intra-operative or post-operative complications were excluded. Furthermore, the use of ClassIntra classification for intra-operative complications was a late decision for this study and as such, data on grade 1 complications, such as bleeding above average managed with routine coagulation or minimal serosal lesion not requiring suturing were not collected.

## Conclusion

In this study, the author adhered to the same structured approach throughout the study period. The SOSURE steps (with the exception of systematic uterine suspension when posterior compartment disease is present) are used widely by most endometriosis surgeons and therefore SOSURE is not a novel technique. However, we believe adherence to this approach and performing high volume of cases may have been contributing factors to the low complication rates seen in our series.

## Supplementary material

Figure SI

Table SI

Table SII

Table SIII

Table SIV
