# Supplementary figures and images for: Intra-operative and post-operative complications of endometriosis excision using the SOSURE approach — A single-surgeon retrospective series of 1116 procedures over 8 years
Source: Facts Views Vis Obgyn. 2024 Sep 30;16(3):325–36. doi: 10.52054/FVVO.16.3.030 (PMC11569428; doi:10.52054/FVVO.16.3.030)

LADN

LPSW

LUSL

UVF

VAG

RECT

RUSL

RPSW

RADN

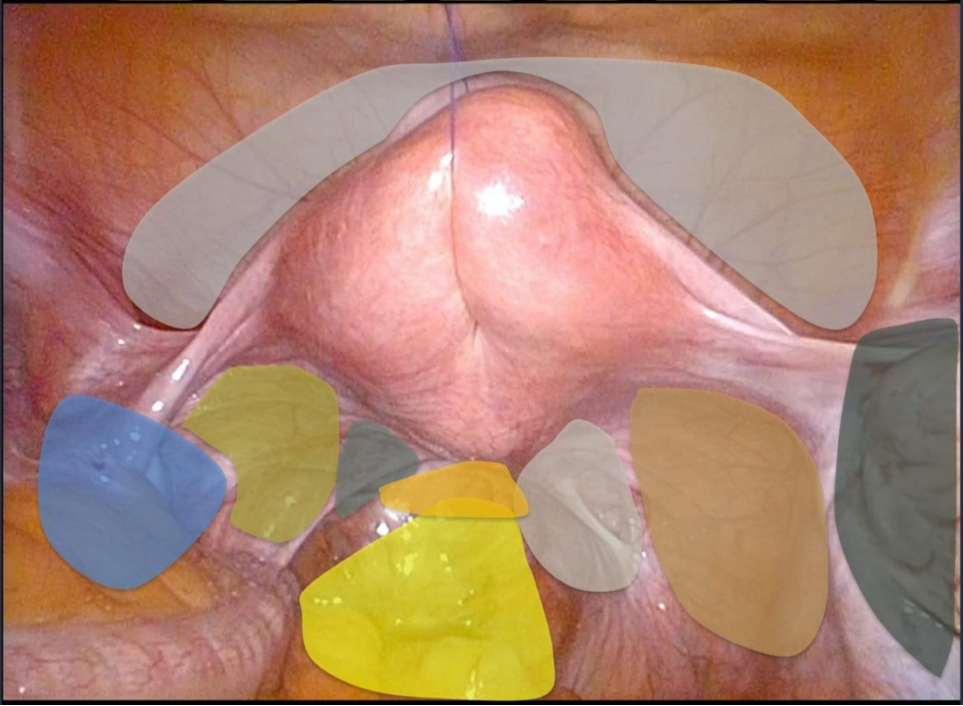

Supplement: Figure SI [file FVVinObGyn-16-325-sf001.pdf]
