# Supplementary material for: Intra-operative and post-operative complications of endometriosis excision using the SOSURE approach — A single-surgeon retrospective series of 1116 procedures over 8 years
Source: Facts Views Vis Obgyn. 2024 Sep 30;16(3):325–36. doi: 10.52054/FVVO.16.3.030 (PMC11569428; doi:10.52054/FVVO.16.3.030)
Supplement: Table SI [file FVVinObGyn-16-325-st001.pdf]

Grade	Definition	Examples
0	No deviation from the ideal intraoperative course	-
1*	Any deviation from the ideal intraoperative course:<br>• Without the need for any additional treatment or intervention<br>• Patient with no or mild symptoms	• Bleeding: Bleeding above average from small-calibre vessel: self-limiting or definitively manageable without additional treatment than routine coagulation<br>• Injury: Minimal serosal intestinal lesion, not requiring any additional treatment<br>• Cautery: Small burn of the skin, no treatment necessary<br>• Arrhythmia: arrhythmia (e.g. extrasystoles) without relevance
2	Any deviation from the ideal intraoperative course:<br>• With the need for any additional moderate treatment or intervention<br>• Patient with severe symptoms, potentially life threatening or potentially leading to permanent disability	• Bleeding: Bleeding from medium calibre artery or vein, ligation; use of tranexamic acid<br>• Injury: Non-transmural intestinal lesion requiring suture(s)<br>• Cautery: Moderate burn requiring non-invasive wound care<br>• Arrhythmia: Arrhythmia requiring administration of antiarrhythmic drug, no hemodynamic effect
3	Any deviation from the ideal intraoperative course:<br>• With the need for any additional moderate treatment or intervention<br>• Patient with severe symptoms, potentially life threatening or potentially leading to permanent disability	• Bleeding: Bleeding from large calibre artery or vein with transient hemodynamic instability, ligation or suture; blood transfusion<br>• Injury: Transmural intestinal lesion requiring segmental resection<br>• Cautery: Severe burn requiring surgical debridement<br>• Arrhythmia: Arrhythmia requiring administration of antiarrhythmic drug, transient hemodynamic effect
4	Any deviation from the ideal intraoperative course<br>• With the need for any additional major and urgent treatment or intervention<br>• Patient with life-threatening symptoms and/or leading to permanent disability	• Bleeding: Life-threatening bleeding with splenectomy; massive blood transfusion; ICU stay<br>• Injury: Injury of central artery or vein requiring extended intestinal resection<br>• Cautery: Life-threatening burn injury by cautery leading to fire requiring ICU treatment<br>• Arrhythmia: Arrhythmia requiring electroconversion, defibrillation or admission to the ICU
5	Any deviation from the ideal intraoperative course with intraoperative death of the patient	-

*We did not record data on Grade 1 intraoperative adverse events
