# Supplementary material for: Intra-operative and post-operative complications of endometriosis excision using the SOSURE approach — A single-surgeon retrospective series of 1116 procedures over 8 years
Source: Facts Views Vis Obgyn. 2024 Sep 30;16(3):325–36. doi: 10.52054/FVVO.16.3.030 (PMC11569428; doi:10.52054/FVVO.16.3.030)
Supplement: Table SII [file FVVinObGyn-16-325-st002.pdf]

Year	Missing notes	Total number of patients	%
2015	12	76	15.8%
2016	3	72	4.2%
2017	0	87	0.0%
2018	0	161	0.0%
2019	0	226	0.0%
2020	3	152	2.0%
2021	0	168	0.0%
2022	0	174	0.0%
