# Supplementary material for: Intra-operative and post-operative complications of endometriosis excision using the SOSURE approach — A single-surgeon retrospective series of 1116 procedures over 8 years
Source: Facts Views Vis Obgyn. 2024 Sep 30;16(3):325–36. doi: 10.52054/FVVO.16.3.030 (PMC11569428; doi:10.52054/FVVO.16.3.030)
Supplement: Table SIII [file FVVinObGyn-16-325-st003.pdf]

Case	Grade 3b complication	Primary procedure	Description of complication
1	Anastomotic stricture	Segmental bowel resection with primary ileostomy	On the day of scheduled reversal of ileostomy, colorectal team noticed significant stricture of the anastomosis site. A decision was made not to reverse and to bring the patient back for a repeat anterior resection. Ileostomy was reversed at a later date.
2	Bowel leak	Partial thickness discoid excision	Presented on day 12 postoperative with spiking temperature and pelvic collection, managed with a laparoscopy, washout and temporary diverting stoma.
3	Bowel leak	Shave of rectovaginal endometriosis	Initially returned to OR for drainage of pelvic abscess. No fecal matter was seen but a small serosal injury identified and decision was to manage conservatively. The patient did not improve post op eratively and a gastrograffin test revealed a leak which was then managed with temporary ileostomy.
4	Pelvic abscess requiring surgical drainage	Shave of rectovaginal endometriosis	Laparoscopic washout of pelvic abscess.
5	Pelvic haematoma requiring surgical drainage	Excision of superficial endometriosis	Large pelvic haematoma was evacuated laparoscopically but no bleeding point was detected
6	Pelvic haematoma requiring surgical drainage	Excision of deep endometriosis	Large pelvic haematoma was evacuated laparoscopically but no bleeding point was detected
7	Rectovaginal fistula	Ultra-low segmental bowel resection with large partial vaginectomy	Presented on day 15 postoperatively with rectovaginal fistula. Managed with a laparoscopy and diverting stoma.
8	Rectovaginal fistula	Shave of rectovaginal endometriosis and hysterectomy	Presented on day 11 postoperatively with rectovaginal fistula. Managed with a laparoscopy and diverting stoma.
9	Exploratory laparoscopy	Partial-thickness discoid excision	Presented to another hospital with abdominal pain and raised inflammatory markers. Had a laproscopy and no abnormalities were detected.
10	Urinary tract fistula	Excision of deep parametrial endometriosis	Presented on day 14 with ureterovaginal fistula, managed with ureteric stents
11	Bleeding from port site	Partial-thickness discoid excision	Significant bleeding from suprapubic port site. CT angiogram showed subcutaneous arterial bleed. Returned to OR for laparoscopy which showed no intra-abdominal bleeding. Subcutanous bleeder diathermied.
12	Haematuria requiring surgical intervention	Stumpectomy, cystoscopy and bladder biopsy and shave of rectovaginal endometriosis	Returned to OR for management of frank haematuria by the urology emergency team. A large clot was seen in the bladder at cystoscopy probably from the biopsy site. Following evacuation of the clot, a bladder defect was found, likely caused by the repeat cystoscopy. Had insertion of ureteric stents and a repair of bladder defect through a Pfannensteil incision by urologist.
13	Intraabdominal bleeding requiring surgical intervention	Shave of rectovaginal endometriosis and hysterectomy	Laparoscopy to control bleeding from the uterine artery and vaginal vault dehiscence
14	Intraabdominal bleeding requiring surgical intervention	Removal of large Gartner's cyst and excision of deep endometriosis from right parametrium	Presented to another hospital with bleeding. Managed laparoscopically.
