# Supplementary material for: Intra-operative and post-operative complications of endometriosis excision using the SOSURE approach — A single-surgeon retrospective series of 1116 procedures over 8 years
Source: Facts Views Vis Obgyn. 2024 Sep 30;16(3):325–36. doi: 10.52054/FVVO.16.3.030 (PMC11569428; doi:10.52054/FVVO.16.3.030)
Supplement: Table SIV [file FVVinObGyn-16-325-st004.pdf]

| Case | Procedure | Converted to | Decision made by | Reason for conversion |
|---|---|---|---|---|
| 1 | Planned ureteric reimplantation | Extended pfannenstiel | Urology team | Urology team did not perform ureteroneocystostomy laparoscopically |
| 2 | Planned ureteric reimplantation | Extended pfannenstiel | Urology team | Urology team did not perform ureteroneocystostomy laparoscopically |
| 3 | Planned ureteric reimplantation | Extended pfannenstiel | Urology team | Urology team did not perform ureteroneocystostomy laparoscopically |
| 4 | Segmental bowel resection | Extension of umbilical incision | Colorectal team | Malfunctioned circular stapler |
| 5 | Repair of enterotomy | Extension of umbilical incision | Colorectal team | Felt a more secure closure can be achieved |
